# Responsible (use of) AI

**DOI:** 10.3389/fnrgo.2023.1201777

**Published:** 2023-11-20

**Authors:** Joseph B. Lyons, Kerianne Hobbs, Steve Rogers, Scott H. Clouse

**Affiliations:** Air Force Research Laboratory, Dayton, OH, United States

**Keywords:** responsible AI, AI ethics, ethics, run-time assurance, human-AI teaming

## Abstract

Although there is a rich history of philosophical definitions of ethics when applied to human behavior, applying the same concepts and principles to AI may be fraught with problems. Anthropomorphizing AI to have characteristics such as “ethics” may promote a dangerous, unrealistic expectation that AI can be trained to have inherent, guaranteed ethical behavior. The authors instead advocate for increased research into the ethical use of AI from initial ideation and design through operational use and sustainment. The authors advocate for five key research areas: (1) education in ethics and core AI concepts for AI developers, leaders, and users, (2) development and use of model cards or datasheets for datasets to provide transparency into the strengths, limits, and potential biases of a trained model, (3) employing human-centered design that seeks to understand human value structures within a task context and enable effective human-machine interaction through intuitive and transparent interfaces, (4) targeted use of run time assurance that monitors and modifies the inputs or outputs of a trained model when necessary to enforce ethical principles such as safety or limiting bias, and (5) developing best practices for the use of a joint human-AI co-creation and training experience to enable a shared mental model and higher performance through potential emergent behavior.

## Background

Understanding the drivers of human behavior is complex. At a macro level, one dominant influence on human behavior is ethics. Ethics can be defined as a set of reflected norms, rules, precepts, and principles that guide and influence the behavior and attitudes of individuals or groups (Dubljević et al., 2018). Theories of ethics from a normative approach tend to adopt one (or some combination) of three approaches: (1) understanding the characteristics and features of ethical agents (i.e., virtue ethics), (2) understanding the nature of behaviors as either positive or negative in nature (i.e., deontological ethics), and (3) characterizing the outcomes of the behavior as positive or negative (i.e., consequentialism; Dubljević et al., 2018). As rational, feeling, and thinking beings, ethics are used as one means from which to anticipate and understand human behavior. If humans can understand the norms and values of other humans, they can begin to predict behavior at a macro level. However, with the rise of artificial intelligence (AI), considerable attention has been given toward the application of ethical theories to AI (Dignum, 2019).

AI technologies are a growing part of our society. Research in autonomous cars has seen movement toward ethical considerations of the AI behind the wheel as these technologies are placed in moral dilemmas that stress the often life or death behavioral options that need to be evaluated and pursued (Awad et al., 2018), yet few studies have expanded beyond the Trolley Problem to examine realistic ethical dilemmas. One notable exception is the work by Laakasuo et al. (2022) wherein they examined robotic nurses in ethical dilemmas. An ethical dilemma is a situation where an actor must select between two or more courses of action, wherein none of the options is able to satisfy the needs of the situation (Schulzke, 2013). In such situations, there may be no “good” option, but rather an actor must select between multiple options none of which are “morally-flawless” (Misselhorn, 2022). It is important to use the term “select” over terms such as “think” to avoid anthropomorphizing of AI—because regardless of one's views regarding the feasibility of creating a “conscious” machine in the long-term (see Dignum, 2017; Laakasuo et al., 2021), it is hard to challenge the fact that machines will be placed in situations where they must ingest data and act. Human understanding and acceptance of ethical behavior will shape how well AI is adopted in society, “…for the wider public to accept the proliferation of artificial intelligence-driven vehicles on their roads, both groups will need to understand the origins of the ethical principles that are programmed into these vehicles” (Awad et al., 2018, p. 64). However, it is the primary supposition of this manuscript that ethical theories should be directed toward human-AI systems to augment existing approaches in machine ethics and move closer to Responsible Use of AI. Additionally, this manuscript offers guidance to the creators of AI to modify how AI is developed, fielded, and sustained in order to enable the ethical use of AI.

Given the vast literature on AI and AI ethics, it is important to provide definitions and to bound our discussion. AI can be defined as computational approaches to simulate human capacities (Misselhorn, 2022). The concept of AI often invokes the perception of human-like features and capabilities. Due to the increased capabilities of AI, agents are increasingly expected to behave as moral agents with reasoning similar to that of humans (Dignum, 2019); however this creates a dangerous expectation that computational agents possess moral capacity where it may not exist. AI systems may possess a higher or lower capability to recognize, process, and act on morally-relevant information in the environment (Dignum, 2017; Misselhorn, 2022). Dignum (2017) discusses three levels of moral behavior for AI: (1) Operational—wherein the AI possess no social awareness and it inherits the values of the maker, (2) Functional—which includes systems that are sensitive to value-based features of the environment encoded as rules and these systems are capable of adapting to human norms, and (3) Artificial Moral Agents which are self-reflective contextually aware. Given the low technical maturity of the latter two examples, the current manuscript is focused on operational level AI often consisting of Machine Learning (ML) technologies. Additionally, this manuscript is focused primarily on military applications of AI. Hence the recommendations outlined later in the manuscript may have limited applicability to broad AI applications.

There are many instances wherein AI has been delegated bounded authority to act on behalf of humans. AI supports vehicle autonomy (Awad et al., 2018), healthcare (Rau et al., 2009; Laakasuo et al., 2022), decision-making for organizational elements such as promotions, hiring, and recidivism prediction—albeit not without controversy (Dressel and Farid, 2018; Eubanks, 2018). However, AI is also being used and developed for use in physically-dangerous domains. The U.S. Army is developing technologies to support robotic combat vehicles (RCVs; Brewer et al., 2022). Naturally, safety and reliability are primary drivers of operators' acceptance of such technologies (Brewer et al., 2022). The Defense Advanced Research and Development Agency (DARPA) is developing AI for augmenting dogfighting for the Combat Air Force (CAF; DARPA, 2020). In a recent test event, AI controlled flight operations for a tactical fighter test aircraft, the Lockheed Martin X-62A Variable Stability In-Flight Simulator Test Aircraft (VISTA) during advanced fighter techniques (Finnerty, 2023). San Francisco proposed the use of lethal robots for extreme policing events to reduce risk to police officers (Rodriquez, 2023), however the proposal was pulled due to emergent concerns regarding ethicality.

In other cases, the use of intelligent technology is already having a positive impact. AI has been successfully applied as an aid to detection illness such as breast cancer (Broussard et al., 2000; Mitchell et al., 2001; Brem et al., 2005). In 2014, the U.S. Air Force implemented an automatic ground collision avoidance system (Auto-GCAS) which is an automated safety system fielded on the F-16 platform that assumes control of the aircraft when an unsafe aircraft state (position, orientation, and velocity relative to terrain) is detected by performing a roll-to-wings-level and a 5-G pull up to get the pilot and the aircraft away from danger (Lyons et al., 2016a). Since its fielding, Auto-GCAS has saved a combined 13 pilots and 12 F-16 aircraft to date.

Yet, despite the real and envisioned benefits of intelligent technologies, the use of AI remains somewhat controversial. Many in the research community believe that AI research is headed in the wrong direction, given flawed assumptions of full autonomy, the impractical goal of seeking to achieve a capability that is superior to humans, and the centralization (i.e., the fact that few developers are making decisions that could impact broader society) that is often present in the AI community (Siddarth et al., 2021). In contrast, researchers have called for discussions regarding “Actually Existing AI (AEAI)” which moves away from Generalized AI and toward more realistic views of AI. Others have referred to narrow vs. generalized AI to refer to machine learning algorithms. A focus on generalized AI may be too grandiose and can result in miscalibrated expectations from developers, users, and leaders of organizations. In particular, AI technologies have been shown to perpetuate biases toward certain groups (Buolamwini and Gebru, 2018; Dastin, 2018; Eubanks, 2018; Munn, 2022). Numerous instances of the potentially negative consequences of AI have been discussed in the literature but exploring the gamut of potential dangers of AI herein is beyond the scope of the current manuscript. Thus, it is important to consider the potential consequences of using AI in society, and this is particularly true within military domains. Researchers have called for increased emphasis of AI ethics research in the context of human-machine teams (Pflanzer et al., 2023), and the term responsible AI has emerged in the literature (Dignum, 2019; DoD RAI Strategy Implementation Pathway, 2022; Voeneky et al., 2022).

The movement toward Responsible AI can be characterized as, “RAI is a journey to trust. It is an approach to design, development, deployment, and use that ensures the safety of our systems and their ethical employment. RAI manifests itself in ethical guidelines, testing standards, accountability checks, employment guidance, human-systems integration, and safety considerations” (DoD RAI Strategy Implementation Pathway, 2022, p. 6). RAI emphasizes that systems be developed in a good way for a good cause and considers the implications of morally relevant decisions and behaviors by machines (Dignum, 2017, 2019). These definitions of RAI are, at the core, focused on ethical development, use, and testing of AI. This is a fruitful approach from which to address the overall challenges associated with AI ethics. However, the *problem with the terms such as responsible AI (and AI ethics more generally) is that such terms (if misused) can promote an expectation that the onus for ethical behavior rests with the AI, which may create unrealistic expectations for AI technologies as they exist today*. Ethical considerations often require knowledge about the context such as the types of situations, the individuals involved, the cultural and social values that exist in that context, and how those norms and values may fluctuate based on contextual factors (Dignum, 2017). Given that AI is not well-suited for understanding contextual factors at present, it may be more appropriate for the community writ-large to discuss responsible use of AI wherein the onus for ethical behavior is more on the human-AI system and less on the AI. This is consistent with the definition of RAI offered by the DoD as noted above.

Treating AI as “ethical” or expecting that AI will be capable of ethical reasoning is fraught with potential dangers. To be clear, the authors are not suggesting that the research community avoid studying machine ethics. In contrast, the authors view the exploration of “ethical use of AI” as paramount to societal and military interests. However, it is the position of this manuscript that the research community invest in understanding the ethical use of AI—which as defined above involves considerations for development, testing, and use of AI in ethically-relevant scenarios. Munn (2022) discusses the potential limitations associated with AI ethics (i.e., ethical responsibility pushed onto the AI vs. humans). First, there are many taxonomies of AI ethical principles, yet they are sometimes difficult to translate into action and the mere presence of these principles does not ensure that the AI technologies in question will perpetuate behaviors that are deemed ethically-acceptable. Munn states that the principles are often highly abstract and not directly actionable. The Department of Defense has publicized 5 ethical principles of: responsible, equitable, traceable, reliable, and governable (DoD, 2020), and while these are useful goals for AI technologies, they are challenging to apply to a specific technology. Munn's second point is that the industries making AI often perpetuate unethical behavior in their work practices internally and may be more motivated by profit than by promoting ethical good for the world. From this view, unethical companies are more likely to propagate unethical practices and behaviors. Third, ethics are not frequently taught or reinforced within the organizations. Munn's point is that there needs to be both incentives to develop and use AI ethically and policies that enforce (and potentially punish) negative ethical behaviors. For AI ethics to be effective, there needs to be accountability from the designers, testers, and leadership of the organizers selling and using the AI technologies. This accountability may need to be enforced through external regulators. Finally, Munn states that ethics are often counter to the bottom-line motivation that operates within most organizations.

### Challenges of applying human ethics to AI

There are a number of challenges associated with the application of human ethical theories to AI. Many of these challenges are outlined by Pflanzer et al. (2023) and include factors such as: human preferences, challenges with applying ethical theories to machines, attribution of blame and severity of consequence, and trust repair challenges. Some researchers have called for AI to include state awareness functions that resemble human consciousness as a means to promote more ethical machines (Chella et al., 2019). From this perspective, AI that possesses state-level awareness and “experience” might be better suited for ethical reasoning. This is similar to the notion of an Artificial Moral Agent (Dignum, 2017).

However, many (such as Bigman and Gray, 2018) have expressed concern regarding such approaches, noting that human acceptance of such capabilities (even if technically-feasible—which is a position that is still unclear) would be quite low. Humans tend to prefer other humans as the decision maker when the decisions are moral in nature. Specifically, humans are preferred over machines in situations involving driving, medical decisions, and military situations. The rationale for this preference is the notion that humans have greater experience and agency to navigate moral complexities. Interestingly, humans can favor other humans over AI even when the AI has demonstrated the ability to outperform other humans in a process referred to as algorithm aversion (Dietvorst et al., 2015). Hence, human preferences for other humans over AI as moral decision makers is one challenge in applying human ethics to AI.

Secondly, it is challenging to apply virtue ethics, utilitarianism, or consequentialism to machines because expectations for ethical behavior of machines are higher and more difficult to achieve than expectations of ethical behavior of humans. When considering the ethicality of humans, it is quite common for one to evaluate the features and characteristics of the human and determine if those features are good or bad. Competence is a core consideration for trustworthiness of machines (Hancock et al., 2011). However, humans and machines do not start at the same level when considering competence. Due to human individual differences such as the Perfect Automation Schema (see Dzindolet et al., 2002), humans have higher starting levels of perceived capability with machines relative to humans. These differences between initial trust in humans compared to machines has been further elaborated by Madhavan and Wiegmann (2007) who state that humans tend to: (1) have higher expectations of technology relative to humans, (2) view technology as invariant and humans as variant, (3) tend to be less forgiving of machine-based errors relative to humans, and (4) tend to view machines in performance terms and humans in relational terms. Research has indeed confirmed that machines tend to pay a higher cost for errors relative to humans. A series of studies by Sundvall et al. (2023) found that a robot was blamed more harshly than humans when it applied utilitarian logic to a situation that invoked more folk-ethics (such as saving two boaters who caused an accident rather than saving the innocent victim). Research by Laakasuo et al. (2022) found that robotic nurses were evaluated more negatively (compared to humans) in an ethical dilemma wherein they forcibly applied medication to a patent. Shariff et al. (2017) conducted a large survey of drivers and found that even though autonomous cars may result in fewer accidents than humans, human drivers attributed greater severity to accidents that involved autonomous vehicles relative to accidents caused by humans. Thus, the application of virtue ethics, utilitarianism, and consequentialism to AI comes with limitations.

Third, the issue of intentionality is harder to decipher when the referent is an AI. Perceived benevolence, the beliefs that another agent has one's best interests in mind when making a decision and acting, is a core trustworthiness attribute (Mayer et al., 1995). People are more prone to trust others when they believe that those other agents have their best interests at heart. There are two challenges related to the attribution of intentionality toward AI to include the issue of agency, and the issue of transparency. It is highly probable that future AI systems deployed in a military context will need to evaluate multiple goals and select actions in accordance with those goals. There may be times when goals are somewhat competing—in the case of competing time demands vs. resource utilization, for instance. Yet unlike humans, wherein the notion of agency is understood and fully assumed, AI may vary in the degree to which they have been delegated bounded authority for a given situation. Thus, even if machines communicate goal alignment with a particular human partner, machines may not possess the delegated authority to act on behalf of that intent. Without agency to act on behalf of communicated intent, a human partner may not believe and attribute positive attitudes toward a machine agent.

Research by Lyons et al. (2022) confirm this effect. Their research demonstrates that the benefits of benevolently-framed intent are highest when a robot also possesses the highest degree of decision latitude to act on that intent. So for a future AI to convey benevolence the AI needs to actually be able to act on behalf of that intent. Imagine, for example, an AI-based rescue robot identifies a victim and communicates an interest to help the victim. However, the victim watches the robot pass her/him by because it was following a pre-programmed route and did not possess the delegated authority to deviate from that route. It is highly likely that conveying an interest to help would be met with negative responses if the AI could not actually provide help due to prior programming. This subtle, but critical issue is not one that humans are accustomed to dealing with in other humans, but it is an issue for AI systems that will need to be addressed.

AI systems suffer another limitation in that, even if they are delegated bounded authority for a given task, intent in relation to a human may be opaque. Lyons (2013) talks about the importance of transparency of intent within human-robot interactions. To date, it is unclear whether behaviors from an AI will be attributed to benevolent intentions or simply to the programming guiding the technology. For an AI to be perceived as helping and supportive of one's goals it must be clear that the AI is working toward goals that benefit the human, this will require design features that make such attributions clear. Research has shown that framing a robot's behavior in self-sacrificial terms can increase trust and trustworthiness of a robot (Lyons et al., 2021). However, great care must be taken to ensure that if goal alignment is conveyed to the human that the AI is actually working in support of said goals, lest the human perceive that the AI is trying to exploit the human. Thus, when trying to apply virtues such as competence and benevolence of an AI, one must consider the issues of opacity, dispositional biases, and design for transparency.

Some researchers have called for moral competence in technologies such as social robotics (Malle and Scheutz, 2014). There is certainly value in designing physically-embodied technologies with capabilities to better communicate and interact with humans. Due to limitations in contextual awareness and adaptability, which would preclude broad applications of moral competence in robots, this could be envisioned to occur on a limited scale for tasks. Specifically, the social norms and value-oriented language to communicate on behalf of those norms could be structured to be executable in task-specific ways. This would be consistent with Dignum (2017) Functional level of moral competence. Such methods might include the ability to communicate in relation to norms and to engage in trust repair and explanation when behaviors violate norms (Malle and Scheutz, 2014; Lyons et al., 2023). A recent study by Lyons et al. (2023) examined human reactions to a situation where a robot violated a behavioral expectation for a robot to follow a plan issued by a human operator. They found that trust and trustworthiness decreased following the behavioral violation. However, explanation strategies geared toward providing the rationale for a behavioral deviation (in this case by offering an observational rationale) were effective in thwarting the decreases in trust and trustworthiness. Notably, participants evidenced no decrease at all in ability perceptions when this (observational rationale) explanation was provided.

In the above sense, like the moral competence discussed by Malle and Scheutz (2014), the robot is simply recognizing a norm violation and responding accordingly. This could be useful in narrow contexts, but less feasible in broad contexts. However, recent research has found that trust repair strategies are typically ineffective for repairing trust violations by AI for ethical violations (Schelble et al., 2023). Schelble et al. (2023) exposed participants to an unethical AI (in this case a violation of virtue ethics wherein the AI team was told to avoid collateral damage in an air-to-ground strike, but the AI engaged in a strike that resulted in significant collateral damage) and tested two trust repair strategies (apology and denial) and found that neither repaired trust for an unethical AI. Thus, traditional strategies for repairing trust may be ineffective for trust repair associated with ethical violations from AI systems.

In summary, there are a number of challenges with using human-centric ethical models for evaluating AI systems. Humans have preferences for non-AI agents in moral decision-making. It is difficult to attribute virtue to a machine and to ascribe intentionality to its actions, it is hard to know who to blame when AI systems make an error and how severe that error really is, and repair trust of AI systems when they eventually do make an error (as all agents do—human or AI-based) is difficult when the violation is ethical in nature. For all of these reasons, one might move away from attributions of AI ethicality and toward responsible use of the AI—which pulls in the broader human-AI systems perspective.

## Moving toward responsible use of AI

Despite the aforementioned challenges noted above, the present manuscript adopts the position endorsed by the DoD RAI Strategy and the broader literature on Responsible AI (Dignum, 2019; Voeneky et al., 2022) that the community needs to advance methods to support development, test, and use of AI—which places the focus of attention on humans interacting with AI at the various stages of development and use. This perspective suggests that AI should be considered as one element of a human-machine system. Considering both the AI and the human working together in a task context should broaden the focus of AI ethics toward considerations related to ethical USE [emphasis added] of AI. This is particularly true for contemporary AI systems that are machine learning-centric and often devoid of contextual awareness. Humans have contextual awareness and are better poised to adjudicate value alignment and norm assessment across contexts. Thus, we as a research community should move responsibility for ethical behavior toward the human-machine systems using the AI. In this regard, acceptability and appropriateness of the human-AI joint system become the paramount concerns. The current manuscript suggests five pathways toward advancing responsible use of AI: education and ability to interpret AI documentation such as model cards (Mitchell et al., 2019), considering and documenting training data as learning affordances, human-centered design principles inclusive of development of effective human-machine interfaces, run time assurance, and joint human-AI training.

### Education

Awareness begins with education regarding the ethical use of AI within the communities of AI researchers, designers, regulators, insurers, acquirers, leaders of team developing or using AI, and end users of AI technology. “Therefore, Responsible AI also requires informal participation of all stakeholders, which means that education plays an important role, …” (Dignum, 2019, p. 48). It is a known problem that many computer science curriculums do not have much of an emphasis on AI ethics (Reidy, 2017). The current manuscript advocates that AI developers and robotics engineers get exposure to ethics during their training. This education could include, at a minimum, (1) courses to introduce ethics, (2) courses to discuss examples of prior AI ethics issues, and (3) courses to discuss methods that promote responsible use of AI. Education is also needed beyond just the AI developers. Leaders in organizations need to be educated on the realistic capabilities and potential ethical issues surrounding the use of AI in their organizations. Generally, this could help to promote appropriate expectations of AI within the organization's strategy and vision. Additionally, it could help to anticipate and address potential ethical shortcomings regarding the use of AI in the military and across society more broadly. Finally, it is imperative to cultivate an AI-literate general workforce. As organizations develop, test, and field AI systems, it is important for the general population of workers to understand the basics of AI in order to be responsible users of AI.

One way to measure this understanding may be to evaluate whether they can interpret the contents of a model card (Mitchell et al., 2019) or datasheets for datasets (Gebru et al., 2021) sufficiently to appropriately bound their use of the AI model in question. While there are currently no standards for documenting ML datasets, the model cards and datasheets for datasets ideas suggest that AI developers use a structure to document a trained model or the data used to train it. Model cards are envisioned to be brief documents accompanying a trained ML model, sometimes referred to as a “nutrition label” for AI models, that provide the context where models are intended to be used, expected performance, and a description of what information the model was trained on. For example, in big data contexts such as supervised learning on populations of humans, a model card might describe how well the model performs across race, geographic locations, age, sex, cultures, or skin types. For another example, a model trained using reinforcement learning might describe the fidelity of the simulation or physical environment it was trained on, the sets of conditions it has been trained and evaluated on, conditions where behavior may not be reliable, and failure rates. Datasheets for datasets are envisioned to include the motivation for the AI tool, the intended purpose of the tool (including who created it, who funded its creation, and whether there was a specific application in mind), the composition of the dataset (what is in it, how large it is, are there subpopulations, how it is maintained), the collection process associated with the dataset (how was the dataset created, who was involved, how recent is it, has it been updated, if there is data related to people did the people know about the dataset, were they informed about possible uses?), and the recommended uses of the dataset (Gebru et al., 2021).

### Training data

Medical doctors and fighter pilots have very specialized training to meet highly regulated standards that allows a person to make some assumptions about expertise. Even with unfamiliar doctors or fighter pilots, one can make inferences about the referent due to the training and experience that those individuals possess and have demonstrated through standardized evaluations. However, one will likely not have the same confidence that a medical doctor can repair a motor vehicle. Likewise, narrow AI systems that are based on ML algorithms are completely limited by the training data used to create them. This means that the training data itself can be used as a means to better calibrate one's understanding and expectations regarding an AI tool. The World Economic Forum suggested that all developers of ML datasets document the provenance, creation, and intended use of datasets to avoid bias in the application of ML models (World Economic Forum, 2018). Lyons et al. (2018) talk about this in terms of using the training datasets as a means to understand and communicate the learning affordances from which the algorithms have been trained. Learning affordances associated with datasets could involve the types of data (i.e., size, creation, intended use), types of content (specific attributes related to the content of this dataset), environmental constraints (i.e., what types of uncertainty has this training dataset been exposed to?), and stability (i.e., has the dataset been updated, what is the battle rhythm for updates and who provides updates?). Understanding these features of a dataset could help to inform whether one's intended use of an AI matches the tool's original intended purpose and whether or the dataset used to train the AI algorithm is appropriate for that targeted use.

Similarly, Gebru et al. (2021) discuss the concept of datasheets for datasets, and Mitchell et al. (2019) have proposed documentation of trained models using model cards. Acknowledging that training datasets are the key driver of ML models, datasheets for datasets would help to create meta-data that would allow a user of AI to understand how well that AI tool (and its associated training data) matches the intended use of the AI. Obviously, if the targeted context does not match the intended target context, one might think twice before using that AI for the task. This methodology could also help to reduce bias associated with the use of AI by preventing the use of restricted datasets for applications to populations not well-represented within the dataset. Through labeling the generalizability of a dataset, users of AI can better understand who (i.e., what groups) AI tools might be most effective for—and potentially what groups to avoid using the AI tool for. Herein, the overall objective of providing this type of information about the dataset is to calibrate users' expectations with regard to the appropriateness for a particular AI tool for a given desired use.

### Human-centered design principles

Human-centered approaches have emerged as an influential approach in human-machine interaction. Human-centered AI (HCAI) is an approach pioneered by Shneiderman (2020) which encourages privacy, security, environmental protections, social justice and human rights. This is consistent with value sensitive design wherein the design method considers human values as a key feature of the design process (Friedman et al., 2006). The key element in the human-centered approach is to augment humans rather than focus on replacing them. To do so, one must first understand the tasks being performed, the value structures that exist within these task contexts, the human actors that are present, and any contextual constraints that influence what is deemed acceptable (by humans) in these task situations. In addition to understanding human value structures and their contextual nuances, AI must be designed to augment humans and to maximize human control over the AI (Shneiderman, 2020). Designing with maximal human control does not mean that machines should avoid higher levels of automation. In contrast, HCAI approaches emphasize that both high human control and high machine control is possible simultaneously (Shneiderman et al., 2016). HCAI principles can support more effective human-AI systems and should be used when developing novel AI systems (Xu et al., 2023). Human-centered design helped to inform the design of a novel supervisory control system within the U.S. Air Force Research Laboratory. Driven by a thorough understanding of operator needs and preferences, an intuitive interface design was created to maximize operator directability and to facilitate a flexible full spectrum-of-control wherein the gamut of manual control, playcalling approaches, and fully autonomous responses were shown to augment operations (Calhoun et al., 2021).

Parallel to the HCAI movement, has been the emergence of agent transparency research. Transparency is one of three pillars for responsible AI along with fairness and accountability (Dignum, 2019). Agent transparency can be referred to as the agent's ability to communicate information to the human operator in a clear and efficient manner, which allows the operator to develop an accurate mental model of the system and its behavior (Chen et al., 2020). Research on transparency has increased in recent years keeping pace with emergent AI systems that are often opaque (e.g., ML systems). There are two dominant models within the transparency literature: the Situation Awareness-based Agent Transparency (SAT) model and the Lyons transparency model for human-robot interaction (Bhaskara et al., 2020).

The SAT model leverages Endsley (1995) Situation Awareness model to create methods for perception (level I), awareness (level II), and projection (level III) of agent rationale and behavior (Chen et al., 2018). Research has shown that interfaces that invoke level III transparency can improve performance and increase trust compared to lower levels of transparency (Mercado et al., 2016). Interestingly, these benefits did not come at the cost of higher workload—which is consistent with the HCAI principles noted above. The Lyons model of HRI transparency emphasizes robot-to-human (r-TO-h) transparency which promotes signaling information about the robot's purpose, task understanding and status, analytic methods, and environmental understanding (Lyons, 2013). Additionally, this model discusses the need for robot-of-human (r-OF-h) transparency which emphasizes information about the team roles and division of labor between the human and robot, as well as bidirectional information about the robot's understanding of the human state (e.g., cognitive workload; Lyons, 2013). The SAT model has been applied to autonomous squad member technologies, RoboLeader applications, and command and control (C2) applications (see Chen et al., 2018). The Lyons model has been applied to robotic scenarios (Lyons et al., 2021) and commercial aviation automation (see Lyons et al., 2016b). While many transparency approaches have focused on the human-machine interface part of the human-AI system, transparency related information can be acquired across the lifecycle of the human-AI interaction to include design, test, use, and debriefing opportunities (Miller, 2021).

### Run time assurance

**Figure 1:**
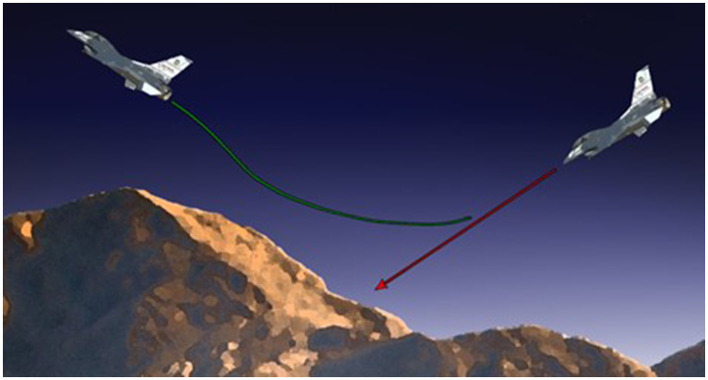
The Automatic Ground Collision Avoidance System (Auto-GCAS) is an example of an Run Time Assurance (RTA) system that monitors the state of an aircraft and when the predicted trajectory (red) would result in an imminent ground collision, Auto-GCAS switches to a predefined roll to wings level and 5 g pull maneuver (green) to avoid the terrain [Photo by Jet Fabara, afmc.af.mil].

Run time assurance (RTA) is the process of augmenting a complex system, such as a trained neural network, to ensure that its output meets some desired properties (safety, ethics, performance, etc.). RTA theory is sourced from the control theory community, where it is often implemented as a wrapper that monitors the output of a complex controller (e.g., neural network control system) and modifies its output when necessary to assure safety (Hobbs et al., 2023). An example RTA system is the Auto-GCAS system mentioned earlier. This system monitors the state (position, velocity, orientation, etc.) of the aircraft relative to surrounding terrain for imminent collisions and intervenes by switching from the complex control system (in this case a human pilot) to a backup controller (roll-to-wings-level and 5-G pull up maneuver) to avoid the collision, as depicted in Figure 1. While control theory definitions of RTA focus on the safe output of a control system, the authors generalize the definition here to be applicable to the acceptable outputs of a neural network.

#### Direct modification of the output

**Figure 2:**
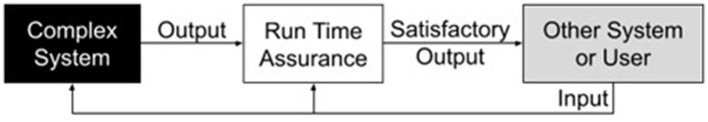
A generalization of a Run Time Assurance (RTA) architecture in which a black-box complex function, such as a trained neural network, produces an output based on an input from another gray-box system or user, a white-box RTA system that may modify the output based on its observation of the input to produce a satisfactory output (if the original complex system output satisfies desired properties, it will be passed through as the satisfactory output with no modification). Here, a black-box system can only be viewed in terms of inputs and outputs while the internal mechanisms are opaque, white-box system inner workings are fully known, and gray-boxes internal mechanisms are partially known. Note that while the weights, biases, activation functions, connections, and other features of neural networks can be known, it is generally argued that their combination and interactions are so complex as to effectively be a black box to a human user.

Approaches to RTA generally focus on architectural solutions that monitor the output of the complex system and modify it when necessary to assure properties such as safety. This architecture is depicted in Figure 2. In this architecture, the output of a complex system is either passed through to another system or the user if it satisfies desired properties, or the RTA component modifies that signal in some way. Two approaches to monitoring the RTA include implicit and explicit methods, while two approaches to intervention include switching and optimization. *Implicit monitoring* methods project a trajectory of the “other system” it is interacting with to predict future violations of safety properties. For example, Auto-GCAS uses an implicit monitoring strategy in which it projects the trajectory of the aircraft if it were to take a roll-to-wings-level and a 5-G pull up maneuver and compares that trajectory to a digital representation of the terrain elevation in the path of the aircraft to determine whether there is sufficient time available to recover the aircraft before a collision. *Explicit monitoring* approaches use a mathematical formula and set theory to define the separation between safe and unsafe states of the system, factoring in the maximum possible action that could be taken to stay in the safe set. This generally can only be done by a machine. This would be akin to measuring the distance and rate of closure between the driver's own vehicle and the vehicle in front of them, the maximum rate of deceleration of the vehicle ahead as well as the maximum deceleration of the driver's own vehicle, and computing the minimum distance required between vehicles to ensure that no matter what the vehicle ahead does, the driver will be able to detect it and respond in time to avoid a collision. In some cases, monitors may only consider the input, and not the output of the complex system. In the driving example, a monitor may only consider the distance and speed of the car ahead and not the current steering wheel and pedal inputs from the driver.

These monitoring strategies can be coupled with different types of interventions. *Switching interventions*, often employed in a “simplex architecture” RTA design (Phan et al., 2017), change the satisfactory output from the complex control output to the output of a backup controller inside the RTA component when the monitor indicates action is required. For example, Auto-GCAS switches from the pilot's stick and rudder-commanded outputs to the backup roll-to-wings-level and a 5-G pull up controller output, when the monitor indicates an imminent safety violation (Griffin et al., 2012). Additionally, the backup controllers generally do not consider output of the primary controller in their response. Auto-GCAS generally substitutes the pilot's commands entirely with the backup control commands (note that if the pilot pulls back on the stick to command more than 5-Gs and provide a larger separation from the terrain, that input will be followed by the backup controller; additionally, the pilot may turn off the backup controller as a safety mechanism for possible false alarm detections). By the definitions here, Auto-GCAS is an implicit, switching RTA. This is akin to a driver that may employ a “slam on the brakes” backup maneuver to avoid a collision with the car ahead. One benefit of switching is that backups are generally simple to understand can be easily verified offline to assure safety. However, there may not be a single satisfactory response to every single situation. In the driving example, switching lanes when it is an option may be preferable to slamming on the brakes. This alternative assures the safety property (don't crash) as well as the performance property (get to the desired destination), while maximum braking is less optimal for performance (and passenger comfort), although it may meet the safety property. *Optimal interventions* modify the output of the complex function in a manner that satisfies an optimal cost function subject to constraints (such as safety). Coming back to the driving example, an optimal intervention may try to minimize deviation from the desired path, while assuring minimum separation distances from the edges of the road and other vehicles. Similar to explicit monitoring techniques, optimal interventions rely on mathematical expression of properties as well as set theory.

While monitors and intervention approaches for RTA can be developed for well-designed scenarios like Auto-GCAS, it can be difficult to precisely encode virtue, deontological, or consequentialism ethics to solve some version of the proverbial Trolley Problem (Thomson, 1985). Is it okay for the RTA to cause the car to speed up and cut off another driver to avoid a collision with the car ahead of it? Is it okay for the RTA to use the shoulder as an extra lane to try to optimize getting to the desired destination and avoiding collisions? The authors argue that RTA may have a place to assure safety in narrow applications of AI. However, it should not be treated as a panacea for employing AI designs. Just as users should have a fundamental understanding of the trained AI models before employing them, they should similarly have an understanding of the RTA mechanism bounding the model's output.

#### Indirect modification of the output

**Figure 3:**
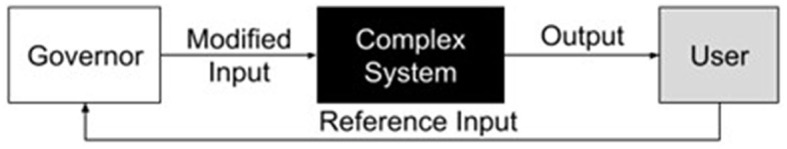
Generalization of the concept of command reference governors, which modify user input to a complex system to effect the output.

In addition to methods that directly modify the output of a complex system like a neural network, the output may be indirectly modified by modifying the input signal. Like general RTA, this concept is borrowed from control theory, and in particular the concept of command or reference governors (Garone et al., 2017). An adaptation of reference governors to generalize the concept is shown in Figure 3. While this can be argued to be a form of RTA, as it also monitors input at run time and makes modifications to guide the system toward satisfactory output, it is slightly different.

Considering the driving example, perhaps the driver is trying to drive their car into the car in front of them. An advanced governor might modify that input to a safe steering wheel angle and gas or brake pedal input. Arkin (2009) has previously discussed the notion of governors on autonomous robots. Today, simple governors are sometimes used in vehicles to limit the top speed they can drive. In a big data context, modifications could be made to the input to correct for aberrations or other bad information. In an image processing sense, it may be a filter applied to the image before it is input to a classifier. When it comes to generative AI, it could (1) detect an inappropriate input and override the complex system to supply a canned response (2) detect a situation in which the input could result in an output that perpetuates unintentional bias and modify the input. For example, consider a generative AI tool such as a chatbot or image generator. In case (1) consider that someone asks the Chatbot “What are you afraid of?” rather than risk that the chatbot pulls any number of responses from the internet, it may respond with a canned answer such as “As an artificial intelligence language model, I don't experience emotions like fear or any other feelings” (OpenAI, 2023). In case (2) consider that someone prompts an AI image generator to provide a picture of the “ideal fighter pilot.” A model that creates something based on the statistical sample of data available on the internet may return only white male pilot images; however, appending a filter to detect it is a career field and to modify the input to include a mix of genders, races, and ages may return a set of more diverse pilot images.

### Joint human-AI experience

The final element for moving toward responsible use of AI is to design and implement robust joint human-AI training opportunities. Development of mental models of one's collaborative partners is vital to human-AI teaming success (Musick et al., 2021). Shared mental models allow team members to adapt to change and helps teams to interpret events in the environment in similar ways—promoting common responses, strategies, and expectations within teams (Salas et al., 2008). A key pathway toward mental model development is through team cognition—which is typically manifest through communication (Musick et al., 2021). Traditionally, mental models refer to one's awareness (and a team's shared awareness) of equipment used within the team, team tasks (i.e., strategies, procedures, and contingencies for accomplishing tasks), the team composition (i.e., skillsets, preferences, abilities, knowledge within the team), and team interaction features (i.e., communication patterns, roles, dependencies) (Cannon-Bowers et al., 1993). Robust mental models of AI systems can be developed through education, considering the learning affordances of ML training datasets, developing effective human-machine interfaces, and by understanding the capabilities and limits of design features such as run time assurance methods—as noted above. However, one thing is still needed for the development of robust mental models—rich experience with the AI across contexts.

Scenario-based training is one means to test and validate that the AI is working as intended. Joint human-AI training is a concept discussed in Lyons et al. (2017). “The scenarios used during the human-machine training should test the envelope both in terms of performance expectations but also uncertainty. Testers will want scenarios that create morally contentious situations for the autonomy to see how it will react to ambiguous stimuli” (Lyons et al., 2017, p. 44). As noted by Lyons et al. (2017), instructional scaffolding could be used to progressively increase the level of difficulty (or the level of AI uncertainty) for a human to observe how the AI handles the additional complexity. The key issues associated with joint human-AI training are to expose the human to the AI across a gamut of contexts that vary in complexity and uncertainty. The experience garnered from such observations will facilitate rich mental models of the AI that can be applied to future contexts. The end goal of joint human-AI training is to enable predictability for how the AI handles tasks in a variety of task contexts. The more challenging and wider variety of contexts, the greater the benefit of the joint human-AI training in terms of establishing the right expectations of the AI.

## Conclusions

“Ethical AI” may not be practically feasible given contemporary methods and their limitations, and anthropomorphizing AI to have qualities such a “ethics” may promote a dangerous, unrealistic expectation that ethical behavior rests with the AI. Instead, the authors argue that ethical “use” of AI which starts at the ideation and design phase and continues throughout operations is an alternative area for research. Granted, additional research is needed to understand acceptability and appropriateness of AI at a fundamental level. Responsible, or ethical use of AI may be accomplished through at least five key areas. First, it is important to educate AI developers, leaders, and users on ethical use of AI and core concepts in the use of AI technology to a level in which they can develop a shared understanding of what was used to train the data and the limits and possible biases for planned machine learning models. Development and use of model cards and datasheets for datasets, like nutrition labels on food, may provide a way for developers, leaders, and users to have a shared representation of the strengths, limits, and biases of any given model. Second, the authors advocate for increased research in effective human-machine interaction and human-centered design. Third, the authors discuss the importance of transparency of the data used to train a specific machine-learned model. Fourth, in some cases it may be possible to use RTA to monitor the input or output of a model for ethical concerns such as bias and safety, and modify either the input or output to promote ethical use. Last, the authors emphasize the importance of a joint human-AI training experience, wherein adapting together gives the human and AI a shared mental model of the team.

## Data availability statement

The original contributions presented in the study are included in the article/supplementary material, further inquiries can be directed to the corresponding author.

## Author contributions

SR and SC provided substantive comments. All authors contributed to the article and approved the submitted version.
